# Supplementary material for: Endothelial sensing of AHR ligands regulates intestinal homeostasis
Source: Nature. 2023 Aug 16;621(7980):821–9. doi: 10.1038/s41586-023-06508-4 (PMC10533400; doi:10.1038/s41586-023-06508-4)
Supplement: Supplementary file 1 — Reporting Summary [file 41586_2023_6508_MOESM1_ESM.pdf]

nature portfolio

Corresponding author(s): Chris Schiering; Benjamin Wiggins

Last updated by author(s): 09/07/2023

# Reporting Summary

Nature Portfolio wishes to improve the reproducibility of the work that we publish. This form provides structure for consistency and transparency in reporting. For further information on Nature Portfolio policies, see our Editorial Policies and the Editorial Policy Checklist.

## Statistics

For all statistical analyses, confirm that the following items are present in the figure legend, table legend, main text, or Methods section.

| n/a | Confirmed | |
|---|---|---|
| ☐ | ☒ | The exact sample size (*n*) for each experimental group/condition, given as a discrete number and unit of measurement |
| ☒ | ☐ | A statement on whether measurements were taken from distinct samples or whether the same sample was measured repeatedly |
| ☐ | ☒ | The statistical test(s) used AND whether they are one- or two-sided<br>*Only common tests should be described solely by name; describe more complex techniques in the Methods section.* |
| ☒ | ☐ | A description of all covariates tested |
| ☐ | ☒ | A description of any assumptions or corrections, such as tests of normality and adjustment for multiple comparisons |
| ☐ | ☒ | A full description of the statistical parameters including central tendency (e.g. means) or other basic estimates (e.g. regression coefficient) AND variation (e.g. standard deviation) or associated estimates of uncertainty (e.g. confidence intervals) |
| ☐ | ☒ | For null hypothesis testing, the test statistic (e.g. *F*, *t*, *r*) with confidence intervals, effect sizes, degrees of freedom and *P* value noted<br>*Give P values as exact values whenever suitable.* |
| ☒ | ☐ | For Bayesian analysis, information on the choice of priors and Markov chain Monte Carlo settings |
| ☒ | ☐ | For hierarchical and complex designs, identification of the appropriate level for tests and full reporting of outcomes |
| ☒ | ☐ | Estimates of effect sizes (e.g. Cohen's *d*, Pearson's *r*), indicating how they were calculated |

*Our web collection on statistics for biologists contains articles on many of the points above.*

## Software and code

Policy information about availability of computer code

| | |
|---|---|
| Data collection | The following software was used for data collection: scRNAseq/bulk RNAseq data - Illumina RTA v2.11.3; flow/spectral cytometry - BD FACSDiva v9 and Cytek Spectroflo v3; Confocal Imaging - Leica LAS-AF v2.7.3.9723 and Zeiss ZEN Black v14.0.27.201; qPCR - BioRad CFX Maestro v1.1; Single cell imaging - PerkinElmer Harmony 4.9 software. |
| Data analysis | Code used for data analysis of scRNAseq dataset included CellRanger v.4.0.0 (demultiplexing, basecall conversion, genome alignment, barcode/UMI counting), blc2fastq v.2.17.1.14 (file conversion), Seurat v.3.2.0 (filtering, clustering, conserved and DE gene analysis), EnrichR web app (gene set enrichment), Pagoda2 v.1.0.8 (overdispersion analysis), and SCENIC v.1.2.4 (regulatory network analysis). ggplot2 v.3.3.3 and ComplexHeatmap v2.2.0 were used for scRNAseq data visualisation. Code used for data analysis of RNAseq datasets (3 mouse datasets, 1 human dataset) included Illumina RTA v.2.11.3 (demultiplexing and basecalling), STAR v.2.2.7a and tophat2 v.2.0.11 (genome alignment for mouse and human datasets respectively), DESeq2 v1.30.1 (normalisation and DE analysis). Flow cytometry data was analysed with FlowJo v10.6 (FlowJO LLC), Confocal data with FIJ v.2.9.0 (ImageJ), and single cell imaging with Harmony v4.9 software (PerkinElmer). Data presentation and statistical analysis performed with GraphPad Prism v9 for non-sequencing data. |

For manuscripts utilizing custom algorithms or software that are central to the research but not yet described in published literature, software must be made available to editors and reviewers. We strongly encourage code deposition in a community repository (e.g. GitHub). See the Nature Portfolio guidelines for submitting code & software for further information.

# Data

Policy information about availability of data

All manuscripts must include a data availability statement. This statement should provide the following information, where applicable:

- Accession codes, unique identifiers, or web links for publicly available datasets
- A description of any restrictions on data availability
- For clinical datasets or third party data, please ensure that the statement adheres to our policy

All sequencing data (murine scRNAseq, murine RNAseq, and human RNAseq datasets) have been deposited in the NCBI gene expression omnibus as a superseries – accession number GSE201789. Mouse genome mm10 sequences were retrieved from retrieved from GENCODE mouse genome (GRCm38), version M23 (Ensembl 98) https://www.gencodegenes.org/mouse/release_M23.html. In SCENIC analysis, RcisTarget was used (https://resources.aertslab.org/cistarget/) and database mm10__refseq-r80__500bp_up_and_100bp_down_tss.mc9nr.feather was downloaded for our analysis. scRNAseq metrics/metadata, conserved and DE gene lists from sequencing experiments, Regulons (generated by SCENIC), genesets that comprise Pagoda2 outputs (Aspects) and input genesets for Pagoda2 overdispersion analysis are all provided as supplementary tables. All other flow cytometry, images and qPCR data are presented within the manuscript. All raw data is provided as source data files accompanying the manuscript.

# Human research participants

Policy information about studies involving human research participants and Sex and Gender in Research.

| | |
|---|---|
| Reporting on sex and gender | N/A |
| Population characteristics | N/A |
| Recruitment | N/A |
| Ethics oversight | N/A |

Note that full information on the approval of the study protocol must also be provided in the manuscript.

# Field-specific reporting

Please select the one below that is the best fit for your research. If you are not sure, read the appropriate sections before making your selection.

☒ Life sciences ☐ Behavioural & social sciences ☐ Ecological, evolutionary & environmental sciences

For a reference copy of the document with all sections, see nature.com/documents/nr-reporting-summary-flat.pdf

# Life sciences study design

All studies must disclose on these points even when the disclosure is negative.

| | |
|---|---|
| Sample size | No statistical methods were used to estimate sample sizes before the study. We based our sample numbers on a combination of preliminary data from pilot studies, previous experience with in vivo and in vitro systems (Schiering et al. Nature 2017, Metidji et al. Immunity 2018), and standards in the field. |
| Data exclusions | scRNAseq thresholding involved excluding cells with abnormally high or low transcripts, and based on high mitochondrial gene content as is standard practice for downstream analysis of this sequencing data. We also excluded doublets, and removed contaminant clusters to improve stringency, as described in the methods. For each BEC and LEC RNAseq, 1 control and 1 experimental repeat were excluded from the original n=4/group based on low Cyp1a1 activation and highly divergent transcriptome (experimental samples), and highly divergent transcriptome only (control samples). |
| Replication | With the exception of scRNAseq due to the nature of the analysis, all experiments were replicated 2-3 times in this study unless otherwise stated. |
| Randomization | Within genotypes, all animals were age and sex matched, and randomized prior to experimentation. When genotype constituted experimental group, mice were age and sex matched as close as possible. Experimental and control cage-matched littermates were used in all mouse experiments with two exceptions: 1) bulkRNAseq experiments where control and experimental mice were both Cre+ from different strains, 2) infection studies where mixing would confound the experiment due to possible enhanced contagiousness of control and experimental groups. For infections of WT mice, mice were randomly assigned to experimental or control groups before the experiments, cage densities matched, and rack positions remained as close as possible. Experimental procedures always involved processing control and experimental samples in a random order, rather than by condition. |
| Blinding | Blinding was not used in this study as the researchers must predetermine genotype before the study in order to obtain equal group sizes, minimize animal usage, achieve appropriate randomization and prevent infection-based artifacts (see above). |

# Reporting for specific materials, systems and methods

We require information from authors about some types of materials, experimental systems and methods used in many studies. Here, indicate whether each material, system or method listed is relevant to your study. If you are not sure if a list item applies to your research, read the appropriate section before selecting a response.

## Materials & experimental systems

| n/a | Involved in the study |
| --- | --- |
| ☐ | ☒ Antibodies |
| ☒ | ☐ Eukaryotic cell lines |
| ☒ | ☐ Palaeontology and archaeology |
| ☐ | ☒ Animals and other organisms |
| ☒ | ☐ Clinical data |
| ☒ | ☐ Dual use research of concern |

## Methods

| n/a | Involved in the study |
| --- | --- |
| ☒ | ☐ ChIP-seq |
| ☐ | ☒ Flow cytometry |
| ☒ | ☐ MRI-based neuroimaging |

## Antibodies

Antibodies used

For flow cytometry:
anti-mouse CD105 BV786, clone MJ7/18, BD Biosciences cat #564746
anti-mouse CD11b PE-Cy7, clone M1/70, BD Biosciences cat #552850
anti-mouse CD11c AlexaFluor647, clone N418, Biolegend cat #117312
anti-mouse CD19 PE, clone 6D5, cat #115508
anti-mouse CD24 AF700, clone M1/69, Biolegend cat #101836
anti-mouse CD3 FITC, clone 17A2, Biolegend cat #102405
anti-mouse CD3 PE-Cy5, clone 17A2, Biolegend cat #100274
anti-mouse CD31 AlexaFluor647, clone MEC13.3, Biolegend cat #102516
anti-mouse CD4 BV650, clone RM4-5, Biolegend cat #100545
anti-mouse CD45 BV510, clone 30-F11, BD Biosciences cat #563891
anti-mouse CD45 PerRCP-Cy5.5, clone 30-F11, Biolegend cat #103132
anti-mouse CD64 PE-Dazzle594, clone X54-5/7.1, Biolegend cat #139320
anti-mouse CD74 AlexaFluor488, clone In1/CD74, Biolegend cat #151005
anti-mouse CD8α BV570, clone 53-6.7, Biolegend cat #100740
anti-mouse CD80 PE, clone 16-10A1, Biolegend cat #104708
anti-mouse CD86 AlexaFluor700, clone GL-1, Biolegend cat #105024
anti-mouse EpCAM BV605, clone G8.8, Biolegend cat #147303
anti-mouse ICAM-1 PeRCP Cy5.5, clone YN1/1.7.4, Biolegend cat #116124
anti-mouse Ly6C BV711, clone HK1.4, Biolegend cat #128037
anti-mouse Ly6G BV510, clone IA8, Biolegend cat #127633
anti-mouse I-A/I-E BV421, clone M5/114.152, Biolegend cat #107632
anti-mouse NK1.1 BV786, clone PK136, Biolegend cat #108749
anti-mouse PD-L1 BV605, clone 10F.9G2, Biolegend cat #124301
anti-mouse PDPN PE-Cy7, clone 8.1.1, Biolegend cat #127412
anti-mouse TCRβ FITC, clone H57-597, Biolegend cat #109215
anti-mouse VCAM-1 BV786, clone 429 (MVCAM.A), BD Biosciences cat #740865
anti-mouse TruStain Fx (blocking antibody anti-CD16/32), clone 93, Biolegend cat #101320

For confocal microscopy:

Primary antibodies:
AlexaFluor488-conjugated rabbit anti-mouse GFP, polyclonal, ThermoFisher cat #A-21311
Goat anti-mouse VEGFR2, polyclonal, R&D Systems cat #AF644
Rat anti-mouse Lyve-1, clone 223322, R&D systems cat #MAB2125
Rabbit anti-mouse Lyve-1, polyclonal, Abcam cat #Ab14917
Rat anti-mouse CD31, clone MEC13.3, BD Biosciences cat #553370
Goat anti-mouse ESM1, polyclonal, R&D Systems cat #AF1999
Rat anti-mouse MADCAM-1, clone MECA-367, Biolegend cat #120702

Secondary antibodies:
AlexaFluor555-conjugated Donkey anti-goat IgG, ThermoFisher cat #A-21432
AlexaFluor647-conjugated Donkey anti-goat IgG, ThermoFisher cat #A-21447
AlexaFluor647-conjugated Donkey anti-rabbit IgG, ThermoFisher cat #A-31573
AlexaFluor488-conjugated Donkey anti-rat IgG, ThermoFisher cat #A-21208
AlexaFluor594-conjugated Donkey anti-rat IgG, ThermoFisher cat #A-21209
Secondary antibodies:
AF555-conjugated donkey anti-goat IgG, ThermoFisher cat #A-21432
AF594-conjugated donkey anti-rat IgG, ThermoFisher cat #A-21209

Single-cell imaging:

Primary antibodies:
anti-human phospho-Rb, clone D20B12, Cell Signalling Technology cat #8156
anti-human Rb, clone 4H1, Cell Signalling Technology cat #9309
anti-human p27 kip1, clone D37H1, Cell Signalling Technology cat #3688
anti-human E2F1, clone EPR3818(3), Abcam cat #ab179445

Secondary antibodies:
AF488-conjugated goat anti-mouse IgG, ThermoFisher cat #A-11001
AF568-conugated goat anti-rabbit IgG, ThermoFisher cat #A-11004

Validation

All antibodies are commercially available, and were validated for specificity and application by manufacturers listed above (Biolegend, BD Biosciences ThermoFisher, R&D Systems, Cell Signalling Technology, Abcam). Antibodies for flow cytometry were titrated in digestion-matched small intestinal samples before use by assessing expression within CD31+ endothelial cell populations, compared to CD45+ and CD31-CD45- non endothelial controls. Antibodies for imaging were used at concetrations suggested in previous published methodologies (https://www.nature.com/articles/nprot.2016.092), or titrated in-house.

See following links for manufacturer validation for flow cytometry antibodies:
anti-mouse CD105 BV786, clone MJ7/18, BD Biosciences cat #564746 https://www.bdbiosciences.com/en-us/products/reagents/flow-cytometry-reagents/research-reagents/single-color-antibodies-ruo/bv786-rat-anti-mouse-cd105.564746
anti-mouse CD11b PE-Cy7, clone M1/70, BD Biosciences cat #552850 https://www.bdbiosciences.com/en-us/products/reagents/flow-cytometry-reagents/research-reagents/single-color-antibodies-ruo/pe-cy-7-rat-anti-cd11b.552850
anti-mouse CD11c AlexaFluor647, clone N418, Biolegend cat #117312 https://www.biolegend.com/en-us/products/alexa-fluor-647-anti-mouse-cd11c-antibody-2703
anti-mouse CD19 PE, clone 6D5, cat #115508, https://www.biolegend.com/en-us/products/pe-anti-mouse-cd19-antibody-1530
anti-mouse CD24 AF700, clone M1/69, Biolegend cat #101836, https://www.biolegend.com/en-us/products/alexa-fluor-700-anti-mouse-cd24-antibody-12790
anti-mouse CD3 FITC, clone 17A2, Biolegend cat #102405, https://www.biolegend.com/en-us/products/fitc-anti-mouse-cd31-antibody-120
anti-mouse CD3 PE-Cy5, clone 17A2, Biolegend cat #100274, https://www.biolegend.com/en-us/products/pe-cyanine5-anti-mouse-cd3-antibody-21198
anti-mouse CD31 AlexaFluor647, clone MEC13.3, Biolegend cat #102516, https://www.biolegend.com/en-us/products/alexa-fluor-647-anti-mouse-cd31-antibody-3094
anti-mouse CD4 BV650, clone RM4-5, Biolegend cat #100545, https://www.biolegend.com/en-us/products/brilliant-violet-650-anti-mouse-cd4-antibody-7634
anti-mouse CD45 BV510, clone 30-F11, BD Biosciences cat #563891, https://www.bdbiosciences.com/en-us/products/reagents/flow-cytometry-reagents/research-reagents/single-color-antibodies-ruo/bv510-rat-anti-mouse-cd45.563891
anti-mouse CD45 PerRCP-Cy5.5, clone 30-F11, Biolegend cat #103132, https://www.biolegend.com/en-us/products/percp-cyanine5-5-anti-mouse-cd45-antibody-4264
anti-mouse CD64 PE-Dazzle594, clone X54-5/7.1, Biolegend cat #139320, https://www.biolegend.com/en-us/products/pe-dazzle-594-anti-mouse-cd64-fcgammari-antibody-12424
anti-mouse CD74 AlexaFluor488, clone In1/CD74, Biolegend cat #151005, https://www.biolegend.com/en-us/products/alexa-fluor-488-anti-mouse-cd74-clip-antibody-16473
anti-mouse CD8α BV570, clone 53-6.7, Biolegend cat #100740, https://www.biolegend.com/en-us/products/brilliant-violet-570-anti-mouse-cd8a-antibody-7377
anti-mouse CD80 PE, clone 16-10A1, Biolegend cat #104708, https://www.biolegend.com/en-us/products/pe-anti-mouse-cd80-antibody-43
anti-mouse CD86 AlexaFluor700, clone GL-1, Biolegend cat #105024, https://www.biolegend.com/en-us/products/alexa-fluor-700-anti-mouse-cd86-antibody-3410
anti-mouse EpCAM BV605, clone G8.8, Biolegend cat #147303, https://www.biolegend.com/en-us/products/pe-anti-mouse-human-cd324-e-cadherin-antibody-9276
anti-mouse ICAM-1 PeRCP Cy5.5, clone YN1/1.7.4, Biolegend cat #116124, https://www.biolegend.com/en-us/products/percp-cyanine5-5-anti-mouse-cd54-antibody-14748
anti-mouse Ly6C BV711, clone HK1.4, Biolegend cat #128037, https://www.biolegend.com/en-us/products/brilliant-violet-711-anti-mouse-ly-6c-antibody-8935
anti-mouse Ly6G BV510, clone IA8, Biolegend cat #127633, https://www.biolegend.com/en-us/products/brilliant-violet-510-anti-mouse-ly-6g-antibody-9121
anti-mouse I-A/I-E BV421, clone M5/114.152, Biolegend cat #107632, https://www.biolegend.com/en-us/products/brilliant-violet-421-anti-mouse-i-a-i-e-antibody-7147
anti-mouse NK1.1 BV786, clone PK136, Biolegend cat #108749, https://www.biolegend.com/en-us/products/brilliant-violet-785-anti-mouse-nk-1-1-antibody-10367
anti-mouse PD-L1 BV605, clone 10F.9G2, Biolegend cat #124301, https://www.biolegend.com/en-us/products/purified-anti-mouse-cd274-b7-h1-pd-l1-antibody-4481
anti-mouse PDPN PE-Cy7, clone 8.1.1, Biolegend cat #127412, https://www.biolegend.com/en-us/products/pe-cyanine7-anti-mouse-podoplanin-antibody-6674
anti-mouse TCRβ FITC, clone H57-597, Biolegend cat #109215, https://www.biolegend.com/en-us/products/alexa-fluor-488-anti-mouse-tcr-beta-chain-antibody-2713
anti-mouse VCAM-1 BV786, clone 429 (MVCAM.A), BD Biosciences cat #740865, https://www.bdbiosciences.com/en-us/products/reagents/flow-cytometry-reagents/research-reagents/single-color-antibodies-ruo/bv786-rat-anti-mouse-cd106.740865
anti-mouse TruStain Fx (blocking antibody anti-CD16/32), clone 93, Biolegend cat #101320, https://www.biolegend.com/en-us/products/trustain-fcx-anti-mouse-cd16-32-antibody-5683

## Animals and other research organisms

Policy information about studies involving animals; ARRIVE guidelines recommended for reporting animal research, and Sex and Gender in Research

| | |
|---|---|
| Laboratory animals | The study used the following mouse strains: Cdh5(PAC)Cre/ERT2Ahrfl/f, Cdh5(PAC)Cre/ERT2Ahrfl/f NuTRAP, Cyp1a1CreR26LSL-eYFP, and Ahr-/-. All mice were bred onto a C57/B6 background. Mice were housed in individually ventilated cages, at ambient temperatures (19-21C), and subjected to a standard 12:12 hour light:dark cycle. Mice were between 6-16 weeks at time of experiments, and subjected to tamoxifen-mediated Cre depletion between 5 and 8 weeks of age. |
| Wild animals | This study did not involve wild animals. |
| Reporting on sex | Male animals were used throughout, except in yersinia pseudotuberculosis infection experiments when a combination of male (Extended Data Fig. 8c, Fig. 3h) and female (Extended Data Fig. 7b, Fig 3g) mice were used. |
| Field-collected samples | This study did not involve samples collected from the field. |
| Ethics oversight | Mouse studies were approved by and in compliance with local AWERB ethics committee as well as UK home office regulations. |

Note that full information on the approval of the study protocol must also be provided in the manuscript.

## Flow Cytometry

### Plots

Confirm that:

☒ The axis labels state the marker and fluorochrome used (e.g. CD4-FITC).

☒ The axis scales are clearly visible. Include numbers along axes only for bottom left plot of group (a 'group' is an analysis of identical markers).

☒ All plots are contour plots with outliers or pseudocolor plots.

☒ A numerical value for number of cells or percentage (with statistics) is provided.

### Methodology

| | |
|---|---|
| Sample preparation | Mouse tissue preparation and staining:<br>Following harvesting and fat removal, small intestine (SI) and colon were cut open longitudinally, and underwent an IEL wash: incubated with IEL wash buffer (IMDM +1%FCS, 5mM EDTA, 10mM HEPES, penicillin/streptomycin, and 2mM DTT) for 20min at 37°C with 200rpm. shaking. Small intestine was washed and vortexed at low speeds in SI PBS (PBS +5mM EDTA+10mM HEPES) 30seconds x 3 times or until clear, and then vortexed at low speed in PBS. Colon was vortexed in PBS once. Both gut tissues were then cut into small pieces and incubated in digestion buffer. All other tissues (BAT, iWAT, liver, lung, kidney spleen) were cut into small pieces and incubated directly in digestion buffer.<br><br>All organs were digested by incubation in Collagenase A digestion buffer (4mls/tissue: HBSS + 20mM HEPES, 10mg/ml Collagenase A (Sigma), 8U/ml Dispase II (Sigma), 50[illegible]g/ml DNase I (Sigma) for 20 minutes at 37[illegible]C with 200rpm shaking. Reactions stopped through addition of 1:1 complete media (IMDM +1%FCS, penicillin/streptomycin, 1x glutamax) and: passing through 100[illegible]m filters (SI, colon, kidney, lung, spleen); debris removal through 2x 1minute 60g centrifugation, harvesting supernatant and then passing through 100[illegible]m filters (liver); or 250g 10-minute centrifugation and careful floating adipocyte fraction removal (BAT, iWAT). After centrifugation (400g, 8 minutes), SI and colon were subjected to 40% Percoll (Amersham) density gradient centrifugation (400g, 8 minutes) to remove debris, while all other tissues underwent resuspension in 0.5ml ACK lysis buffer for 2 minutes and washing to remove erythrocytes. Finally, cells were filtered (40[illegible]m) and counted before downstream analysis.<br><br>Cell suspensions were incubated with anti-mouse CD16/CD32 (TrustainFx, Biolegend), before incubation with live/dead dye (zombie near infa-red, Biolegend), and incubation with surface antibodies. All antibodies and subsequent washes in FACS buffer (PBS + 2% FCS, 2mM EDTA). For EdU detection, Click-iT EdU proliferation kit – pacific blue (ThermoFisher) was used, according to manufacturer's instructions. DAPI nuclear stain added directly <5minutes prior to analysis/sorting. A combination of compensation beads (OneComp/Ultracomp eBeads, ThermoFisher) for antibody controls, and single-stained cells for dyes and fluoresecent proteins were used for controls and for compensation/spectral unmixing.<br><br>Human cell preparation and staining:<br>Primary human umbilical vein endothelial cells (HUVEC; Lonza, UK) obtained from pooled donors were seeded at 60-80% confluency and cultured in Endothelial Cell Growth Medium-2 media (EGM-2 bulletkit; Lonza) supplemented with penicillin/streptomycin(P/S) at 5% CO2 and 37oC. Cells were switched to minimal growth media (EGM-2 Bullet kit basal medium, Lonza; supplemented with P/S) for 24h prior to experimentation.<br><br>Click-iT EdU proliferation kit AlexaFluor647 (ThermoFisher, C10340) was used according to manufacturer's instructions. Briefly, this involved a 1h EdU pulse to cells prior to washing, fixing, DAPI staining, and analysis. |
| Instrument | BD LSRII flow cytometer; Cytek Aurora spectral cytometer; BD Aria Fusion cell sorter. |

| | |
|---|---|
| Software | Data was collected with BD FACSDiva v9 software (BD instruments), or SpectroFlo v3 (Cytek Aurora). All analysis was carried out with FlowJo v10.6 (FlowJo LLC). |
| Cell population abundance | Purity of sorted cells was routinely assessed post-sort, reaching purity of above 95%. |
| Gating strategy | Full gating strategies are shown in Extended Data Fig. 3d, and Extended Data Fig. 5a for flow cytometry and FACS experiments respectively. Gating was set based on following density distributions, verified by comparing known/expected expression levels in BEC and LEC, and compared to non-endothelial populations. |

☒ Tick this box to confirm that a figure exemplifying the gating strategy is provided in the Supplementary Information.
