# Supplementary material for: Endothelial sensing of AHR ligands regulates intestinal homeostasis
Source: Nature. 2023 Aug 16;621(7980):821–9. doi: 10.1038/s41586-023-06508-4 (PMC10533400; doi:10.1038/s41586-023-06508-4)
Supplement: Supplementary file 14 — List of antibodies used for flow cytometry and immunofluorescence experiments. [file 41586_2023_6508_MOESM14_ESM.docx]

## Table S12 – list of antibodies used for flow cytometry and immunofluorescence experiments.

| **Flow Cytometry antibodies** | | | | | |
| --- | --- | --- | --- | --- | --- |
| **Antigen** | **Conjugate** | **Clone** | **Dilution** | **Company** | **Cat #** |
| CD105 | BV786 | MJ7/18 | 1/100 | BD | 564746 |
| CD11b | PE-Cy7 | M1/70 | 1/200 | BD | 552850 |
| CD11c | AlexaFluor647 | N418 | 1/400 | Biolegend | 117312 |
| CD19 | PE | 6D5 | 1/200 | Biolegend | 115508 |
| CD24 | AlexaFluor700 | M1/69 | 1/100 | Biolegend | 101836 |
| CD3 | FITC | 17A2 | 1/200 | Biolegend | 102405 |
| CD3 | PE-Cy5 | 17A2 | 1/200 | Biolegend | 100274 |
| CD31 | AlexaFluor647 | MEC13.3 | 1/100 | Biolegend | 102516 |
| CD4 | BV650 | RM4-5 | 1/200 | Biolegend | 100545 |
| CD45 | BV510 | 30-F11 | 1/200 | BD | 563891 |
| CD45 | PeRCP-Cy5.5 | 30-F11 | 1/200 | Biolegend | 103132 |
| CD64 | PE-Dazzle594 | X54-5/7.1 | 1/200 | Biolegend | 139320 |
| CD74 | AlexaFluor488 | In1/CD74 | 1/100 | Biolegend | 151005 |
| CD8α | BV570 | 53-6.7 | 1/200 | Biolegend | 100740 |
| CD80 | PE | 16-10A1 | 1/200 | Biolegend | 104708 |
| CD86 | AlexaFluor700 | GL-1 | 1/200 | Biolegend | 105024 |
| EpCAM | BV605 | G8.8 | 1/200 | Biolegend | 147303 |
| ICAM-1 | PeRCP-Cy5.5 | YN1/1.7.4 | 1/100 | Biolegend | 116124 |
| Ly6C | BV711 | HK1.4 | 1/400 | Biolegend | 128037 |
| Ly6G | BV510 | IA8 | 1/400 | Biolegend | 127633 |
| I-A/I-E (MHC-II) | BV421 | M5/114.152 | 1/1200 | Biolegend | 107632 |
| NK1.1 | BV786 | PK136 | 1/100 | Biolegend | 108749 |
| PD-L1 | BV605 | 10F.9G2 | 1/200 | Biolegend | 124321 |
| PDPN | PE-Cy7 | 8.1.1 | 1/200 | Biolegend | 127412 |
| TCRβ | FITC | H57-597 | 1/50 | Biolegend | 109215 |
| VCAM-1 | BV786 | 429 (MVCAM.A) | 1/100 | BD | 740865 |
| CD16/CD32  (TruStain FcX) | - | 93 | 1/500 | Biolegend | 101320 |
| **Immunofluorescence antibodies – Primary** | | | | | |
| **Antigen** | **Conjugate** | **Species/Clone** | **Dilution** | **Company** | **Cat #** |
| CD31 | - | Rat, MEC13.3 | 1/400 | BD | 553370 |
| ESM1 | - | Goat, polyclonal | 1/200 | R & D systems | AF1999 |
| LYVE-1 | - | Rabbit, polyclonal | 1/500 | Abcam | Ab14917 |
| LYVE-1 | - | Rat, 222322 | 1/100 | R & D Systems | MAB2125 |
| MADCAM-1 | - | Rat, MECA-367 | 1/400 | Biolegend | 120702 |
| VEGFR2 | - | Goat, polyclonal | 1/100 | R&D systems | AF644 |
| GFP | AlexaFluor488 | Rabbit, polyclonal | 1/500 | Thermo Fisher | A-21311 |
| **Immunofluorescence antibodies – Secondary** | | | | | |
| Goat IgG | AlexaFluor555 | Polyclonal Donkey | 1/500 | Thermo Fisher | A-21432 |
| Goat IgG | AlexaFluor647 | Polyclonal Donkey | 1/500 | Thermo Fisher | A-21447 |
| Rabbit IgG | AlexaFluor647 | Polyclonal Donkey | 1/500 | Thermo Fisher | A-31573 |
| Rat IgG | AlexaFluor488 | Polyclonal Donkey | 1/500 | Thermo Fisher | A-21208 |
| Rat IgG | AlexaFluor594 | Polyclonal Donkey | 1/500 | Thermo Fisher | A-21209 |
